# Academic librarians as navigators of institutional data policies: developing resources to facilitate compliant research data management

**DOI:** 10.5195/jmla.2026.2404

**Published:** 2026-10-01

**Authors:** Michelle Yee, Nicole Contaxis

**Affiliations:** 1 michelle.yee@nyulangone.org, Librarian, Research Data and Metadata Management; Assistant Curator, Medical Library, NYU Health Sciences Library, NYU Grossman School of Medicine, New York, NY; 2 nicole.contaxis@nyulangone.org, Head, Data Sharing and Metadata Management; Assistant Curator, Medical Library, NYU Health Sciences Library, NYU Grossman School of Medicine, New York, NY

**Keywords:** Data management, academic librarians, data policy, federal regulation, educational guidance

## Abstract

**Background:**

Proper research data management requires complying with data policies that ensure the ethical, effective, and efficient collection, storage, and sharing of research data. Medical librarians have made increasing inroads to leading research data management instruction and institutional efforts to comply with federal policies like the NIH Data Management and Sharing Policy. However, there has been less discussion about how medical librarians can support researcher compliance with institutional data policies. After observing patterns in requests for guidance, two data services librarians developed decision-making tools to help researchers identify appropriate local resources for their data sharing and re-use needs.

**Case Presentation:**

The paper describes four projects that were developed with a similar methodology for the purpose of guiding patrons through institutional policies and processes: (1) a decision tree for determining when a data transfer or use agreement is required; (2) a matching tool that identifies appropriate data transfer platforms for specific data characteristics or scenarios; (3) a decision tree for determining whether a researcher needs to seek out Institutional Review Board (IRB) review for a project that uses secondary data analysis; and (4) a flowchart detailing data de-identification processes and institutional tools available for specific data formats. The creation of each resource entailed information gathering from local experts as well as review of internal policies to produce action-oriented guidance to solve common researcher problems.

**Conclusions:**

These efforts demonstrate how librarians can utilize their skills and develop cross-departmental collaborations to help patrons navigate an increasingly complicated data ecosystem, particularly where that ecosystem is informed both by external policies (e.g., policies for de-identification) and internal tools (e.g., data transfer tools). The decision trees have helped researchers make choices about their own data management approaches so that they comply with external and internal policies. These documents also enable stakeholders to identify gaps in institutional policies and resources. The development of these resources has had the additional benefit of strengthening partnerships and positioning librarians as collaborators for policy decisions involving research data.

## BACKGROUND

In 2011, the New York University Health Sciences Library (NYUHSL) established a data services team to centralize research data management expertise and services for a large academic medical center. Beginning as three librarians with additional liaison duties and one staff member, the data services team today has four data librarians and two staff members. The team provides consultations and training for a range of topics, including data management, data sharing, and how to use institutionally supported tools like REDCap (1, 2). Additionally, they built and maintain a data discovery platform called the NYU Data Catalog, which houses metadata for research data and instructions for data access (3). Their work mirrors a trend in health sciences libraries broadly speaking with an increasing number of libraries offering data services and the establishment of the National Center for Data Services (NCDS), a center from the Network of the National Library of Medicine (NNLM) that is meant to teach librarians about providing data services (4). Over time and through these services and tools, the [redacted] team has grown and developed significant expertise in data sharing and data management as well as relationships with researchers and research support personnel across the institution.

Through these relationships with stakeholders, the development of subject matter expertise, and ongoing interactions with the research community, the two members of the Data Services team were made aware of various needs for guidance on implementing data management best practices that aligned with institutional data policies. Institutional data policies may describe what data is considered confidential, impose requirements on tools for storing and transmitting data, and inform security protocols. These institutional policies typically expand on federal policies, such as the Health Insurance Portability and Accountability Act (HIPAA) Privacy Rule, with more specific local requirements.

This paper describes a novel aspect of data management services: helping researchers understand data policies and how to implement those policies within their pre-existing data workflows (e.g., data sharing). To provide this type of guidance, the authors created decision trees as institutional resources. As frequent collaborators with departments like Information Technology (IT), General Counsel, and the Institutional Review Board (IRB), librarians are well-placed to gather information on data policies, engage with stakeholders across the institution, and apply their expertise in data management in the creation of these resources (5). Furthermore, creating decision trees, as opposed to providing time-consuming consultations and referrals to other departments, are a sustainable approach for librarians to address these information needs. The decision trees created in this use case include information on institutional-specific policies, so to be used at other institutions, these resources would need to be revised to match institutionally specific policies. The approach for developing and disseminating this type of resource is generalizable.

### Relevant Policies

Some key US federal policies formed the basis of institutional data policies described in the case report. Main drivers include the Health Insurance Portability and Accountability Act (HIPAA) and the Federal Policy for the Protection of Human Subjects, frequently referred to as the “Common Rule” (6, 7). In order to develop guidance on institutional data policies based on federal policies, librarians needed to understand these federal policies.

The implementation of HIPAA in 1996 established national standards and practices for protecting health information. In particular, the Privacy Rule limits the uses and disclosure of protected health information (PHI) while placing no restrictions on de-identified health information (8). Two broad methods for de-identification are described in the Privacy Rule. The Common Rule predates HIPAA and governs research conducted on human beings, including the roles and responsibilities of Institutional Review Boards (IRB). The Common Rule focuses on ensuring research participant autonomy and regulates how informed consent is obtained from research participants. An example of the Common Rule’s role in data policy is that research data cannot be shared – even if de-identified – if a research participant is not properly consented to that data sharing (9). Both HIPAA and the Common Rule may shape how institutions police appropriate and inappropriate data management practices.

Internal policies can extend these external policies. For example, the local IT department tests and implements technologies that enable HIPAA-compliant transfer of data with PHI. An internal policy requires researchers to utilize these approved tools, thereby ensuring that they comply with federal guidelines as well. Another type of internal policy is the determination of whether sharing a specific dataset would require a Data Use Agreement (DUA) or Data Sharing Agreement (DSA). While HIPAA outlines requirements for sharing identifiable or limited datasets, an institution may decide to require DUAs or DSAs for non-HIPAA protected data if that data is understood to be commercially viable or sensitive for reasons not related to HIPAA or the Common Rule.

## CASE PRESENTATION

Over the years, a number of information needs were expressed to two Data Services librarians at [redacted], including the following: (1) understanding what internal policies and procedures applied to sharing research data, including creating a DUA or obtaining IRB approval; (2) determining which data transfer tools would be appropriate for legally and ethically transferring data that may contain PHI; (3) identifying when IRB approval is needed for a research project that uses secondary or pre-existing data; and (4) understanding what institutional tools or workflows are available for data de-identification. These four cases were chosen for a variety of factors including interest from institutional stakeholders and frequency of library consults around the issue. Each use case represents a situation where non-compliance with institutional policies could put patient or research participant data at risk. Protecting patient and research participant data is a priority shared by many at any academic medical center. This shared goal provides a stable foundation for collaborations to produce informational resources on data policies and research data management best practices. These four scenarios also presented an opportunity to deepen relationships with other research stakeholders like IT or Risk Management.

Examples of each of these use cases will be used throughout our discussion of the generalized approach to creating guidance for data policies.

For each of these use cases, the authors decided to create decision trees, a form of flowcharts. The format is designed to help researchers and research staff walk themselves through each logical step of a problem. As an alternative to dense documentation, this type of resource is meant to highlight the key decision points of any situation (e.g., whether data was obtained from human subjects data or not) and the ramifications of that decision (e.g., de-identifying human subject data prior to sharing). One of the two data librarians would take the lead for a specific decision tree with the other consulting and providing feedback. Nicole Contaxis led efforts for the decision trees on internal policies for data sharing and tools for data transfer. Michelle Yee led efforts for the decision tree on de-identification and secondary data analysis.

### Approach

The authors created decision trees to provide researcher guidance by following a similar five step methodology, as illustrated in Figure 1. This method developed organically and in an iterative fashion as the authors learned how best to engage other stakeholders and collaborators.

**Figure 1:**
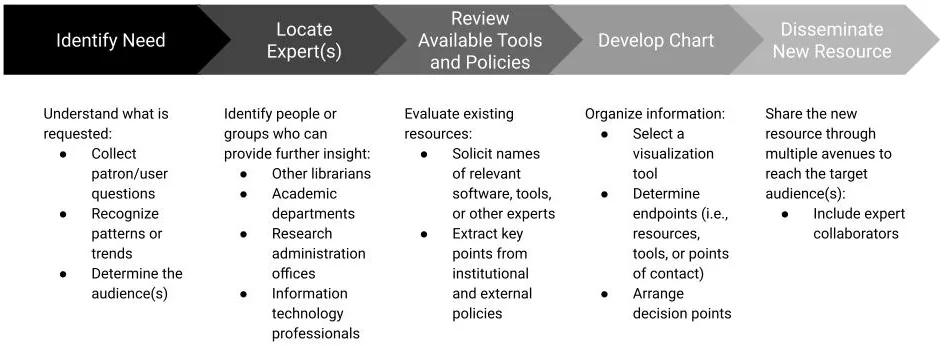
Workflow for decision tree generation

#### Step 1: Identify Need

Information needs surfaced during research data management consultations, within the library ticketing system, or during outreach meetings for open science initiatives like the NYU Data Catalog. For example, the data librarian who eventually led the creation of the data sharing policies flowchart noted that researchers appeared to struggle with these policies when asked to share their data through the NYU Data Catalog. In particular, there was a lack of clarity about when DUAs were needed, when data sharing might be limited by past informed consent paperwork, and when an IRB would need to be involved.

Similarly, data librarians discovered gaps in understanding ethical secondary data use while providing data reference services. Secondary data, pre-existing data that has been collected by someone other than the primary user, may vary in availability and accessibility (10, 11). Through various means, including workshops, individual consultations, the NYU Data Catalog and library guides, the data librarians have connected researchers at all career levels with relevant data and/or search strategies for locating such data. In the course of providing these services, researchers frequently asked whether they would need IRB approval prior to requesting or utilizing secondary data. Typically, they were referred to contacts at the institution’s Human Research Protections (HRP) office and the IRB for further discussion. However, wanting to enable researchers to understand and navigate relevant policies more easily, one of the two data librarians on the project initiated a collaboration with HRP.

In some cases, these information gaps were identified by an existing collaborator or local expert. For example, separate guidance on data transfer tools was produced while creating the data sharing decision tree.

#### Step 2: Locate Experts

After identifying a specific information need, the data librarians located experts in that area within the institution. They sought out stakeholders to provide helpful perspectives from other disciplines and departments. Sometimes stakeholders were also active collaborators, providing feedback on decision trees in development and engaging in dissemination of the final products. Further information on how needs and experts were identified is included in Figure 1.

To produce a decision tree on data sharing policies, it was clear that two kinds of expertise were needed: expertise on the regulatory and policy environment for research data sharing and expertise on researchers’ data sharing practices and culture. Data librarians had significant knowledge of research data sharing practices and culture, but the input of General Counsel was necessary to address the regulatory and policy environment. In particular, the lead librarian collaborated with a lawyer who had experience with data policy and had written the institution’s data retention policy. Similarly, when preparing a decision tree for describing IRB involvement in secondary data analysis projects, the lead librarian sought out an expert from the IRB to interpret the language in federal policies.

To provide guidance on data de-identification processes and tools, a wider array of experts and collaborators was needed. The librarian in charge consulted leaders in various research areas, including directors of clinical informatics and data analysis teams, to compile examples from their own experience in which various types of data required de-identification before being transmitted to another party. This list would eventually be incorporated into the decision tree.

#### Step 3: Review Available Documentation and Tools

Next, both librarians collected information on available tools and policies. This information was used to identify key decision points for shaping the flowchart or decision tree. The process of locating experts, pre-existing policies, and tools may be iterative. A new piece of documentation could point to a new expert not previously consulted or a new expert could point to documentation that the librarians did not previously know existed.

For example, when developing a flowchart on policies related to secondary data analysis projects, the leading librarian located resources created by the United States Department of Health and Human Services (HHS), including the Human Subject Regulations Decision Charts (12), and a self-certification worksheet from the institution’s HRP office that incorporated information from HHS. These materials were used as a starting point for the creation of the flowchart, which eventually underwent several rounds of editing between the librarian in charge and the HRP office.

Sometimes clear documentation did not yet exist. While investigating the suitability of data transfer tools in various contexts, the librarian found little documentation on data transfer tools at the institution. To remedy this issue, there were several in-person meetings between data librarians, the General Counsel, and several members of the institution’s IT departments, including members of the cyber-security, research technology, and desktop support teams.

These meetings began as fact-finding endeavors to relay needs and concerns around current researcher data sharing practices. To facilitate discussions, the lead data librarian presented a list of past instances where researchers needed to transfer data. This allowed her to highlight the need for clearer documentation on policies and technical procedures and to gather information on available tools. The listed common data transfer scenarios included sharing internally, sharing externally, sharing data that included PHI, and sharing data at set intervals during multi-institutional clinical trials.

At the time, the institution had several data transfer tools at hand, including secure file transfer protocol (SFTP), email attachment encryption, and internal cloud workspaces. However, it was not clear which tools were suitable for the transfer of identifiable information in ways that were compliant with federal mandates like HIPAA. This knowledge was documented by IT in their internal security standards, and data librarians worked with IT to locate those standards and then expand them into a decision tree.

#### Step 4: Develop the Flowchart

Finally, flowcharts were created in Microsoft Visio or Canva, tools selected according to the librarian’s preference. Drafts were shared with experts and stakeholders for feedback and revisions. Considering the deeply collaborative nature of these projects, feedback and revisions frequently took several rounds. For example, with the flowchart about IRB approval, staff from HRP assisted in ensuring that the language in the flowchart matched policy as much as possible. For the decision tree on internal policies for research data sharing, General Counsel provided feedback on specific data types or sharing use cases that were missed in prior drafts.

To develop guidance for data de-identification, librarians explored several options on how best to organize the information by referring to external resources from HHS and National Institute of Standards and Technology (NIST), as well as input from local experts. The team explored multiple potential starting points for a decision tree: categorization of the data format or modality (e.g., tabular, imaging, unstructured text), specific features of the data source or destination (e.g., cloud-based, controlled access), and characteristics of the data recipient (e.g., external collaborator, institutional affiliates, general public). The most approachable pathway appeared to be linking data formats to de-identification tools and techniques, which eventually formed the basis of the decision tree. Additional screening questions to assess the risk of re-identification were added to the start of the decision tree to help users determine the applicability of the guide.

Two of the four flowcharts are available as supplemental materials. Appendix A contains the flowchart that demonstrates which internal policies apply to specific instances of data sharing and Appendix B contains the flowchart that demonstrates when IRB approval is necessary for data sharing. Flowcharts that discuss internal tools for data transfer and data de-identification are considered confidential.

#### Step 5: Disseminate New Resources

Dissemination is a key final step. Resources can be shared through traditional routes like library guides, newsletters, and workshops. The data sharing and transfer tool flowcharts are included in a subject guide that has been viewed over 1000 times. The collaborative nature of flowchart development provides additional avenues for dissemination. Having been engaged throughout the information gathering and flowchart creation process, stakeholders have been keen to disseminate the flowcharts through their own channels. For example, after collaborating on the development of the data transfer tools decision tree, the IT department used and circulated the flowchart so thoroughly among their own staff that it was eventually sent back to the library as a useful resource that “they should know about”.

## DISCUSSION

Compliance with institutional data policies is embedded within the practice of research data management. These policies, particularly when implemented by multiple departments or offices across an institution (e.g., IT, Human Research Protections), can be difficult to locate, understand, and apply. Librarians can facilitate compliance with these policies by making them easier to understand and implement. With an understanding of research data management, librarians are well-suited to work across departments and provide educational offerings and guidance on how to implement data policy requirements in day-to-day data management practices.

Creating data policy decision trees provides a series of benefits to library patrons, librarians, and the institution. First, the library helps researchers implement research data management practices in ways that comply with institutional policies. This can be indirectly measured by the number of views of associated library guides and the circulation of decision trees within the medical center. Institutions benefit with the reassurance that the potential for non-compliant practices is reduced.

Second, by working with other departments to create these flowcharts, the team established and built upon new partnerships. For example, after developing a flowchart on data transfer tools with IT, the library was invited to contribute to further projects, including devising documentation on data storage to help IT analysts better refer researchers to appropriate technologies. Additionally, in cases where there were insufficient tools, policies, or documentation to complete a decision tree, working with other departments brought these gaps to their direct attention. These interdepartmental partnerships also allowed librarians to showcase their expertise and improve communication about key policies and procedures that impact research data management.

This approach, however, has several limitations and challenges. Although collaboration can strengthen interdepartmental relationships, progress is dependent on collaborators’ priorities and responsiveness. Keeping stakeholders on track can be a challenge, particularly if other demands emerge over the course of the project. An additional challenge is maintenance of these decision trees to reflect changes to policy or available technologies. This is particularly an issue with software and tools; institutions obtain, replace, or end licenses without broadly communicating these changes. Sometimes it is possible to rely on partners in other departments to keep the library up to date. Finally, in the course of developing many of these decision trees, the team encountered a lack of established institutional policies or guidelines. For example, to develop charts for selecting strategies for data transfer or data de-identification, IT leadership had not yet decided which technologies were best to use in which cases. Discussions that were initially scheduled to create the flowcharts thus became meetings for IT leadership to talk through various facets of data sharing and de-identification. While ultimately helpful for the research community, this process can be time-consuming for librarians.

For librarians considering undertaking similar projects to assist researchers with their institution’s data policies, it is vital to be patient but firm with inter-departmental collaborators. For example, a librarian may work with General Counsel on resources for institutional data retention policies. If the partner in General Counsel is pulled away to address a lawsuit, the librarian can set an alert to touch base with their collaborator in a month or two when the higher priority task has been addressed. Taking this type of approach with collaborators can also help build future relationships, as the librarian is able to demonstrate that they are an effective but understanding partner.

Moving forward, the library plans to continue to work with other departments to produce similar guidance to help researchers manage their data in policy-compliant ways. If insufficient resources exist, librarians are then placed to better inform the creation of new policies and or acquisition of new tools. By doing so, librarians can ensure that the needs of both researchers and the institution are met. This approach affirms the library's central role in an academic medical center’s research ecosystem, helping researchers navigate complex data management challenges to contribute to scientific advancement.

## Data Availability

There are no data associated with this article.

## SUPPLEMENTAL FILES

**Appendix A:** Policy Flowchart

**Appendix B:** IRB Approval Flowchart
