# Supplementary material for: Academic librarians as navigators of institutional data policies: developing resources to facilitate compliant research data management
Source: J Med Libr Assoc. 2026 Oct 14;114(4):477–82. doi: 10.5195/jmla.2026.2404 (PMC13670229; doi:10.5195/jmla.2026.2404)
Supplement: Supplementary file 1 [file jmla-114-4-477-s01.pdf]

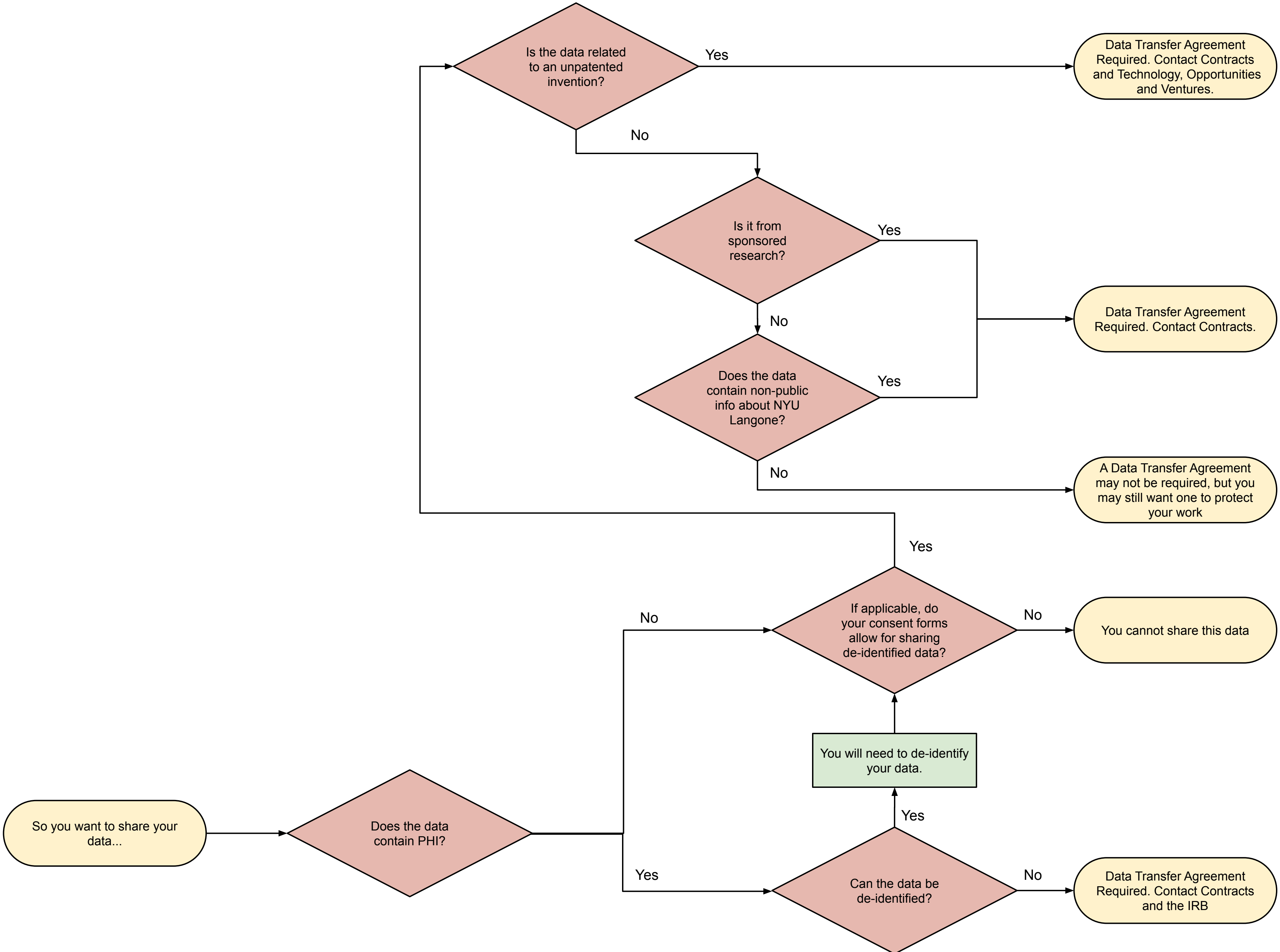
Is the data related to an unpatented invention?
Yes
Data Transfer Agreement Required. Contact Contracts and Technology, Opportunities and Ventures.
No
Is it from sponsored research?
Yes
Data Transfer Agreement Required. Contact Contracts.
No
Does the data contain non-public info about NYU Langone?
Yes
No
A Data Transfer Agreement may not be required, but you may still want one to protect your work
Yes
If applicable, do your consent forms allow for sharing de-identified data?
No
You cannot share this data
No
You will need to de-identify your data.
Yes
So you want to share your data...
Does the data contain PHI?
Yes
Can the data be de-identified?
No
Data Transfer Agreement Required. Contact Contracts and the IRB
