# Supplementary material for: Academic librarians as navigators of institutional data policies: developing resources to facilitate compliant research data management
Source: J Med Libr Assoc. 2026 Oct 14;114(4):477–82. doi: 10.5195/jmla.2026.2404 (PMC13670229; doi:10.5195/jmla.2026.2404)
Supplement: Supplementary file 2 [file jmla-114-4-477-s02.pdf]

Using secondary or existing data: Does your project require Institutional Review Board (IRB) review as human subjects research?

Is your project "research"?

Does it involve a “systematic investigation"?

"Systematic investigation" is an activity that involves a prospective research plan that incorporates data collection, either quantitative or qualitative, and data analysis to answer a research question.

The systematic investigation will involve a predetermined method for studying a specific topic, answering a specific question(s), testing a specific hypothesis(es), or developing theory.

Examples of systematic investigations include, but are not limited to, observational studies, interview (including those that are open-ended) or survey studies, group comparison studies, test development, program evaluation, interventional research.

OR

Is it intended to contribute to generalizable knowledge?

Investigations designed to develop or contribute to generalizable knowledge are those designed to draw general conclusions (i.e., knowledge gained from a study which may be applied to populations outside of the specific study population), inform policy, or generalize findings.

To develop or contribute to generalizable knowledge requires that the results (or conclusions) of the activity are intended to be extended beyond a single individual or an internal program.

NO → Your project does not require IRB review.

YES ↓

Does your project involve human subjects?

Your proposed activity involves “intervention” or interaction with a human subject or uses existing information or biospecimens that were collected from or about living individuals.

NO → Your project does not require IRB review.

YES → Proceed to Page 2

Proceed to Page 2

Your project does not require IRB review.

Updated January 2023 by Data Services at NYU Health Sciences Library

Adapted from the Self-Certification Form created by the NYU Langone Health Institutional Review Board.
For more detailed information or to view decision charts produced by the HHS Office for Human Research Protections, visit Decision Trees for Human Subjects Requirements.

Using secondary or existing data: Does your project require Institutional Review Board (IRB) review?

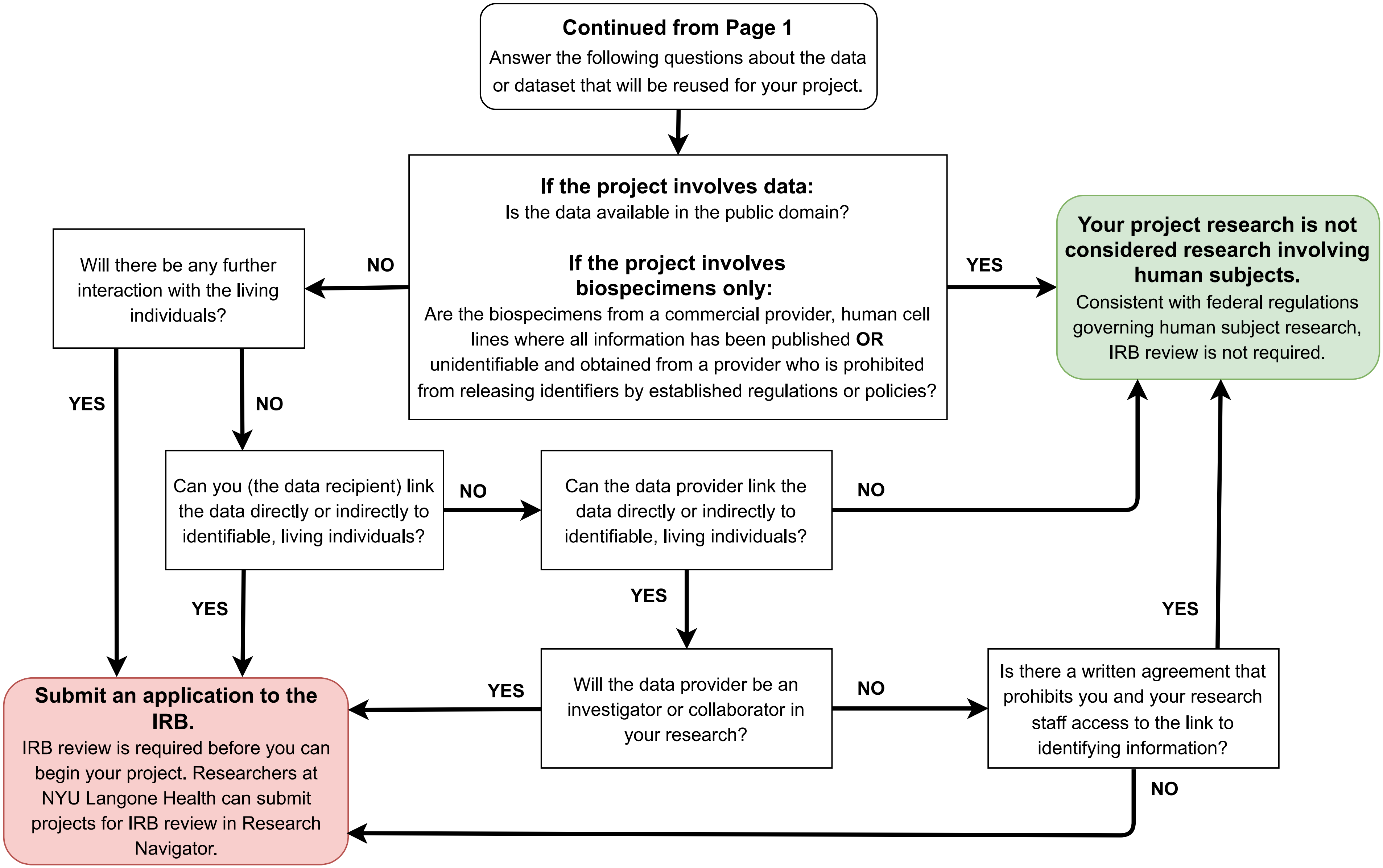

Updated January 2023 by Data Services at NYU Health Sciences Library

Adapted from the Self-Certification Form created by the NYU Langone Health Institutional Review Board.
For more detailed information or to view decision charts produced by the HHS Office for Human Research Protections, visit Decision Trees for Human Subjects Requirements.
